# Thermostability and Immunogenicity of Genotype II Avian Orthoavulavirus (AOaV-1) Isolates from Duck (*Anas platyrhynchos*) and Parrot (*Eclectusroratus*)

**DOI:** 10.3390/v14112528

**Published:** 2022-11-15

**Authors:** Sangeeta Das, Pankaj Deka, Parikshit Kakati, Pubaleem Deka, Mrinal Kumar Nath, Aman Kumar, Arfan Ali, Mihir Sarma, Rofique Ahmed, Sophia M. Gogoi, Arijit Shome, Biswajyoti Borah, Nagendra Nath Barman, Dilip Kumar Sarma

**Affiliations:** 1 Department of Veterinary Microbiology, College of Veterinary Science, Assam Agricultural University, Khanapara, Assam 781022, India; 2 Life Science Education Trust, Bangalore 560064, India; 3 Department of Veterinary Epidemiology and Preventive Medicine, College of Veterinary Science, Assam Agricultural University, Khanapara, Assam 781022, India; 4 Department of Animal Biotechnology, LUVAS, Hisar 125001, India; 5 Department of Poultry Science, College of Veterinary Science, Assam Agricultural University, Khanapara, Assam 781022, India; 6 Department of Veterinary Biochemistry, College of Veterinary Science, Assam Agricultural University, Khanapara, Assam 781022, India; 7 Department of Animal Biotechnology, College of Veterinary Science, Assam Agricultural University, Khanapara, Assam 781022, India

**Keywords:** Newcastle disease, Newcastle disease virus, immunogenicity, thermostability

## Abstract

Newcastle disease (ND) is a highly contagious viral disease of poultry causing significant economic losses worldwide. Vaccination is considered the most reliable approach to curb the economic menace that is ND, but the thermolabile nature of Newcastle disease virus (NDV) vaccination poses a significant threat to its protective efficacy. This study aimed to profile the thermostability of NDV isolates from duck (As/Km/19/44) and parrot (As/WB/19/91) and evaluate their immunogenic potential in chicks. Fusion protein cleavage site (FPCS) and phylogenetic analysis demonstrated the lentogenic nature of both the isolates/strains and classified them as class II genotype II NDV. The characterized NDV isolates were adapted in specific-pathogen-free (SPF) chicks by serially passaging. Biological pathogenicity assessment of chicken-adapted As/Km/19/44 (PSD44C) and As/WB/19/91 (PSP91C) revealed both the isolates to be avirulent with a mean death time (MDT) of more than 90 h and an intracerebral pathogenicity index (ICPI) ranging from 0.2 to 0.4. Both of the NDV isolates displayed varied thermostability profiles. PSD44C was the most thermostable strain as compared to PSP91C and the commercially available LaSota vaccine strain. The immunogenicity of PSD44C and LaSota was significantly higher than PSP91C. Based on these results, it is concluded that NDV isolate PSD44C is more thermostable and immunogenic when administered intraocularly without any adverse effects. Therefore, PSD44C is suitable for further research and vaccine development.

## 1. Introduction

Newcastle disease (ND), caused by *Avian orthoavulavirus 1* (AOAV-1) and formerly designated as *Avian avulavirus 1* (AAvV-1) or *Avian paramyxovirus 1* (APMV-1), is a highly contagious devastating viral disease of poultry worldwide. The causative agent is classified under the genus *Orthoavulavirus*, within the subfamily *Avulavirinae*, of the family *Paramyxoviridae* [1]. It causes significant economic loss in the poultry sector in endemic areas worldwide due to the soaring morbidity and mortality rates associated with such areas. The Newcastle disease virus (NDV) genome is a non-segmented, negative-sense, single-stranded RNA of 15,186 to 15,198 nucleotide long flanking six genes. These genes encode a nucleocapsid protein (N), a phosphoprotein (P), a matrix protein (M), a fusion protein (F), a hemagglutinin-neuraminidase protein (HN) and a large polymerase protein (L) [2]. A wide range of avian species, including both domestic and feral birds, are susceptible to NDV infection [3].

Vaccination remains the most valuable implement for effective prevention and control of ND throughout the world. Most currently available vaccines, particularly the live vaccines, are thermolabile, demanding cold chain maintenance from manufacture to dispensation. These vaccines must be stored at a temperature range of 2–8 °C to remain effective [4], which is challenging under village conditions or remote areas that are often beyond the reach of the cold chain. The disruption of the cold chain leads to the rapid loss of the potency of the vaccine, as well as the administration of ineffective vaccines, resulting in vaccination failure; thisis the biggest contributor to vaccine wastage [5]. The World Health Organization (WHO) estimates that nearly 50% of lyophilized and 25% of liquid vaccines are wasted each year. According to the U.S. National Sciences Foundation (NSF), about 80% of the total cost of vaccination programs is consumed by the “cold chain”. This requirement places a tremendous financial and logistical burden on vaccination programs. The development of thermostable vaccines can greatly ease this problem by facilitating vaccine coverage gains in remote areas and significantly reducing the cost of vaccination programs. Thermostable ND vaccines indeed can be an important contribution in ND control programs, particularly in developing countries.

Addressing the need to develop a thermostable vaccine, several attempts based on reverse vaccinology, the use of chemical stabilizers and the addition of stabilizing adjuvants followed by freeze drying have been made [6,7,8,9]. Numerous studies reported the isolation of low-virulent, thermostable NDV in several species of birds which have been evaluated as vaccine candidates to protect village poultry [10,11,12]. In the present study, lentogenic NDV isolates recovered from duck (*Anas platyrhynchos*) and parrot (*Eclectusroratus*) were adapted to chickens through serial passage. The chicken-adapted As/Km/19/44 (PSD44C) and As/WB/19/91 (PSP91C) was evaluated for thermostability and immunogenicity.

## 2. Materials and Methods

### 2.1. Isolation and Identification of the NDV Isolates from Duck and Parrot

The virus isolates described in this study were isolated under DBT Twinning project tilted “An integrated omics approach to characterize circulating Newcastle disease virus and intervention strategies to control Newcastle disease in North East India”, Department of Microbiology, College of Veterinary Science, Assam Agricultural University, Khanapara, Assam, India from cloacal swab and intestinal content of duck (As/Km/19/44) and parrot (As/WB/19/91), respectively, in 9-day-old specific-pathogen-free (SPF) embryonated chicken eggs (ECE) (Venky’s India Ltd., SPF Eggs Div., Pune, India) through the allantoic cavity route [13]. Briefly, the inoculated eggs were incubated for 3 days at 37 °C and checked daily for embryo viability. Embryos that died within 24 h post-inoculation were discarded and considered dead due to non-specific causes, whereas embryos surviving until the end of incubation were chilled at 4 °C overnight. The allantoic fluid was harvested and hemagglutination (HA) activity was checked by HA assay using 1% (*v/v*) chicken red blood cells (RBC). The identity of the HA-positive allantoic fluid was confirmed by a hemagglutination inhibition (HI) test [13] using NDV-specific hyperimmune serum and RT-PCR amplification of *fusion* (*F*) gene of NDV [14]. The hyperimmune serum was generated in in-house chickens immunized with NDV LaSota vaccine strain (Venkateshwara Hatcheries Pvt Ltd., Maharashtra, India).

#### 2.1.1. Hemagglutination Inhibition (HI) Test

An HI serological test was performed on HA-positive allantoic fluids following standard beta-procedure [13]. Briefly, a serial two-fold dilution of antisera against LaSota was reacted with 4 HA units (HAU) of antigen for 30 min at room temperature. Next, 1% chicken RBC was added and the HI titer was determined after an additional incubation of 30 min. The HI titer is the highest dilution of serum causing complete inhibition of 4 HAU of antigen.

#### 2.1.2. RNA Extraction and RT-PCR Amplification of NDV F-Gene

The presence of NDV in the allantoic fluid was further reconfirmed by RT-PCR. In brief, RNA was extracted from HI-positive allantoic fluids using a Qiagen RNeasy Extraction Kit (Hilden, Germany) following the protocol provided by the manufacturer. The extracted RNA was subjected to two steps RT-PCR using Revert Aid (M-Mulv) (Thermo Fisher). The primer *Fusion*-Forward (5′-TTGATGGCAGGCCTCTTGC-3′) and *Fusion*-Reverse (5′-GGAGGATGTTGGCAGCATT-3′) were used to amplify a 363 bp genome fragment containing a part of *F* gene of NDV [14]. The RT-PCR cycle program was reverse transcription at 50 °C for 30 min, initial denaturation at 94 °C for 5 min, 35 cycles with denaturation at 94 °C for 30 s, annealing at 56 °C for 1 min, extension at 72 °C for 2 min and final extension at 72 °C for 5 min. The amplified RT-PCR products were analyzed by 1.5% agarose gel electrophoresis containing ethidium bromide (0.5 µg mL^−1^) and visualized in an image documentation system.

### 2.2. Molecular Characterization of the NDV Isolates from Duck and Parrot

#### 2.2.1. Sequence Analysis

##### Primer Design

The primers were designed to amplify a 1618 bp full-length *F* gene of NDV. The *F* gene sequences of several NDV isolates were retrieved from the GenBank^®^ (http://www.ncbi.nlm.nih.gov/Genbank/index.html, accessed on 10 September 2022) and multiple sequence alignment was performed using ClutalW (http://clustalw.ddbj.nig.ac.jp/top-e.html, accessed on 10 September 2022). The primers were *Fcds-*Forward (5′-ATGGGCTCCAGACCTTCTACC-3′) and *Fcds-*Reverse (5′-TGYGTTCAYRYTYTTGTRGTGGCTCTCATC-3′).

##### RNA Extraction and RT-PCR Amplification

The total RNA was extracted and full-length *F* gene of NDV was amplified by RT-PCR as previously described. The optimized thermal profile was as follows: reverse transcription at 50 °C for 30 min, initial denaturation at 94 °C for 5 min, 35 cycles with denaturation at 94 °C for 30 s, annealing at 56 °C for 1 min, extension at 72 °C for 1 min 30 s and final extension at 72 °C for 5 min. The amplified products were visualized by electrophoresis on 1% agarose gel stained with ethidium bromide (0.5 µg mL^−1^).

##### Nucleotide Sequencing and Phylogenetic Analysis

Sequencing was performed by outsourcing at 1st BASE DNA sequencing division, Malayasia. The full-length *F* gene sequences were aligned with the BioEdit program using the Clustal W algorithm and analyzed using BLAST. The amino acid motif at the *F* protein cleavage site area of the NDV isolates was also determined.

The phylogenetic and molecular evolutionary analyses were conducted using MEGA X [15]. The nucleotide sequences of NDV isolates from duck and parrot under study were compared with nucleotide sequences of other NDV isolates and reference vaccine strains in the GenBank database using BLAST on the NCBI website. The phylogenetic tree based on full-length *F* gene sequence was constructed by the neighbor-joining method using the sequences under study and from NDV strains with various genotypes published in GenBank. The significance of the deduced phylogenetic tree was verified by bootstrap analysis of 1000 replicates. Nucleotide sequences of commercial vaccine strains LaSota (GenBank acc. AF077761) and B1 (GenBank acc. AF309418) were included in this phylogenetic analysis.

### 2.3. Adaptation of As/Km/19/44 and As/WB/19/91 in Chicken

The NDV isolates from duck (As/Km/19/44) and parrot (As/WB/19/91) were serially passaged in SPF chicks up to six passage levels. Briefly, each of the isolates were inoculated into three 5-day-old SPF chicks via the intraocular and intranasal route. Three days post infection, the chicks were sacrificed and trachea samples were collected. The identity of the virus was confirmed by RT-PCR amplification of *F* gene of NDV [14]. The virus recovered was isolated and used for inoculation in the second passage and so on for a total of six passages. Virus replication in every passage of infected SPF chicks was detected and confirmed by RT-PCR amplification.

### 2.4. Biological Pathogenicity Assessment of Chicken-Adapted PSD44C and PSP91C

The pathogenicity of the chicken-adapted PSD44C and PSP91C was determined on the basis of mean death time (MDT) in eggs and intracerebral pathogenicity test (ICPI) in day-old chicks [13]. MDT was determined by inoculating the isolates in 9-day-old SPF ECE by standard procedures [13,16]. For determining ICPI, 10 1-day-old SPF chicks were inoculated with 50 µLof a 1:10 dilution of infective allantoic fluid with a HA titer 2^5^(Log _2_ 5/50 µL) by intracerebral route. The chicks were observed every 24 h for 8 days and scored as follows: (0) if normal, (1) if sick and (2) if dead. The ICPI was obtained by calculating the mean score per bird per observation over the 8-day period.

### 2.5. Assessment of Thermostability of Chicken-Adapted PSD44C and PSP91C

Seven paired sealed vials containing 1 mLaliquots each of the chicken-adapted PSD44C, PSP91C and LaSota were prepared for two different sets of temperature (40 °C and 56 °C). One pair of each set was left on ice while the other six for each temperature treatment were kept at 40 °C and 56 °C [17]. At time intervals of 5, 10, 15, 30, 45 and 60 min, a pair of vials was removed. All the aliquots at different temperature–time combinations were assayed for HA activity as described earlier. The infectivity of the heat-treated viruses was evaluated by calculating fifty percent Embryo Infectious Dose (EID_50_) [18] using the Reed–Muench method [19]. Briefly, tenfold serial dilutions of each mentioned treatment of both the isolates and LaSota were made in Phosphate Buffered Saline (PBS) and each of these dilutions was inoculated into five ECE. Following 3 days’ incubation, the eggs were harvested for allantoic fluid and confirmed by HA for the presence of the virus. The Reed–Muench method was used to calculate the end point from the results of the HA test on each of the inoculated eggs.

Calculation of proportionate distance (PD) between the two dilutions above and below the 50% end point:

(1) $$\mathrm{PD}=\frac{\left(\%\;\mathrm{infected}\;\mathrm{at}\;\mathrm{dilution}\;\mathrm{immediately}\;\mathrm{above}\;50\%\right)-50\%}{\left(\%\mathrm{infected}\;\mathrm{at}\;\mathrm{dilution}\;\mathrm{immeadiately}\;\mathrm{above}\;50\%\right)-\left(\%\;\mathrm{infected}\;\mathrm{at}\;\mathrm{dilution}\;\mathrm{below}\;50\%\right)}$$

### 2.6. Evaluation of Immunogenicity of the Chicken-Adapted PSD44C and PSP91C

The immunogenicity of chicken-adapted PSD44C and PSP91C was evaluated and compared with commercially available LaSota vaccine strain (Venkateshwara Hatcheries Pvt Ltd., Maharashtra, India). A safety test was performed as per standard protocol before immunogenicity studies [13]. Briefly, ten 5-day-old chicks were inoculated with ten times the single dose each of chicken-adapted PSD44C and PSP91C intraocularly. The chicks were then observed for 21 days to determine the presence of local and/or systemic adverse reactions or death.

#### Experimental Design

A total of sixty 1-day-old SPF chicks were randomly divided into 3 groups: T_1_, T_2_ and T_3_ (*n* = 20). Water and feed were given ad libitum. The chicks of the T_1_, T_2_ and T_3_ groups were immunized against chicken-adapted PSD44C and PSP91C and commercially available NDV LaSota vaccine at 7 days of age via the intraocular route with a standard dose of 10^6^ EID_50_ per chick [13]. HI titers in the blood of experimental chicks were determined before immunization and at 7, 14, 21 and 28 days post immunization (dpi).

### 2.7. Statistical Analysis

The thermal stability of PSD44C and PSD91C at different temperature–time combinations was analyzed statistically by regression analysis and compared with commercial vaccine strain LaSota. In order to determine the half life, nonlinear regression (curve fit) followed by exponential one phase decay analysis was completed. Two-way ANOVA with interaction was also performed along with a critical difference (CD) test for pair-wise comparison of mean serum antibody titer in chicks, keeping time and treatment as factors. Statistical analysis was performed using Graphpad prism 9.3.0.

### 2.8. Ethics Statement

The Institutional Animal Ethics Committee, Assam Agricultural University, Khanapara, Guwahati-781022, Assam reviewed and approved this study (Approval No. 770/GO/Re/S/03/CPCSEA/FVSc/AAU/IAEC/19-20/736 dated 23.12.2019).

## 3. Results

### 3.1. Isolation, Identification and Molecular Characterization of NDV Isolates from Duck and Parrot

**Figure 1:**
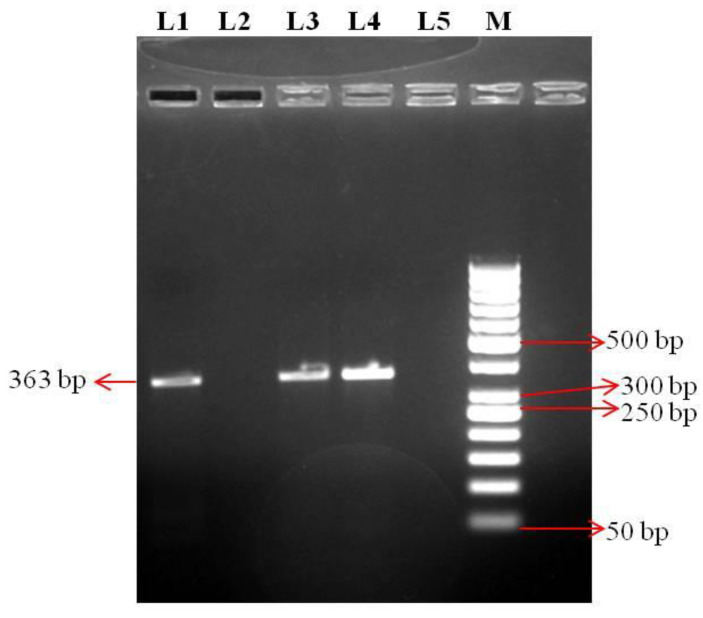
Confirmation of the Newcastle disease virus (NDV) isolates through PCR. PCR carried out with *F* gene specific primers. Lane 3 and Lane 4 show 363 bp *F* gene amplification product of duck NDV isolate (As/Km/19/44) and parrot NDV isolate (As/WB/19/91), respectively; Lane 1, positive control (ND LaSota vaccine); Lane 2, negative control and Marker (M), a 50 bp DNA ladder (Thermo Scientific, Waltham, MA, USA).

The harvested allantoic fluid of the NDV isolates from duck (As/Km/19/44) and parrot (As/WB/19/91) gave a positive reaction to the HA test and the hemagglutinating activity was inhibited by NDV-specific hyperimmune serum in the HI test. A 363 bp fragment covering a part of *F* gene of NDV was successfully amplified in the HI-positive allantoic fluids by RT-PCR (Figure 1).

**Figure 2:**
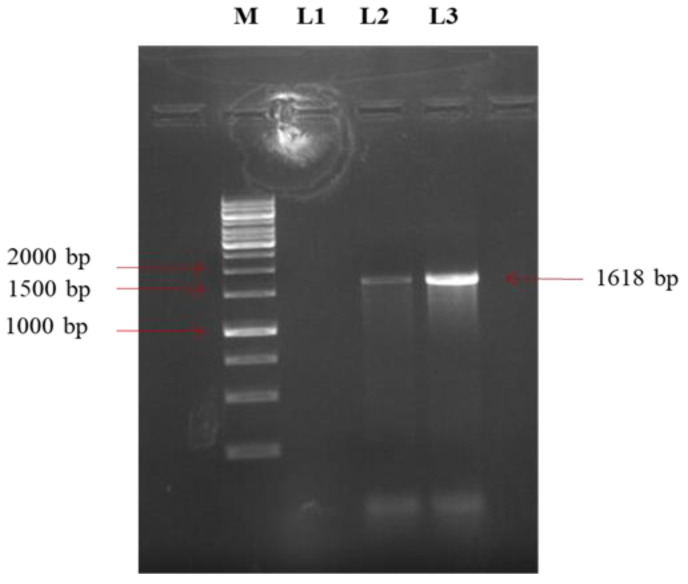
PCR analysis of the NDV isolates. PCR carried out with full-length *F* gene-specific primers. Lane 2 and Lane 3 show 1618 bp *F* gene amplification product of duck NDV isolate (As/Km/19/44) and parrot NDV isolate (As/WB/19/91), respectively; Lane 1, negative control and Marker (M), a 1 kb DNA ladder (Thermo Scientific, Waltham, MA, USA).

**Figure 3:**
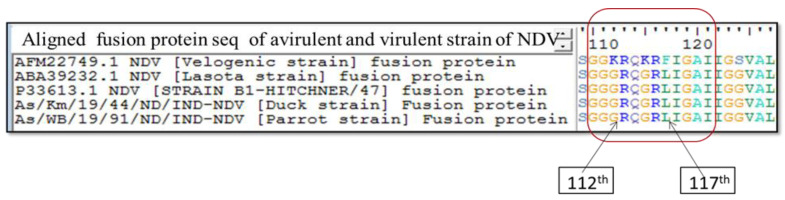
F protein cleavage site analysis for pathotyping of Newcastle disease virus isolates from duck (As/Km/19/44) and parrot (As/WB/19/91).

Further, 1618 bp full length *F* gene of both the isolates was amplified with the designed primers (Figure 2). Molecular pathotyping was conducted based on the amino acid residues of the F0 protein cleavage site motif. The full-length *F* gene sequences showed that both the isolates shared the cleavage site motif ^112^GRQGRL^117^ with an amino acid residue leucine (L) at position 117, indicating its lentogenicity (Figure 3).

**Figure 4:**
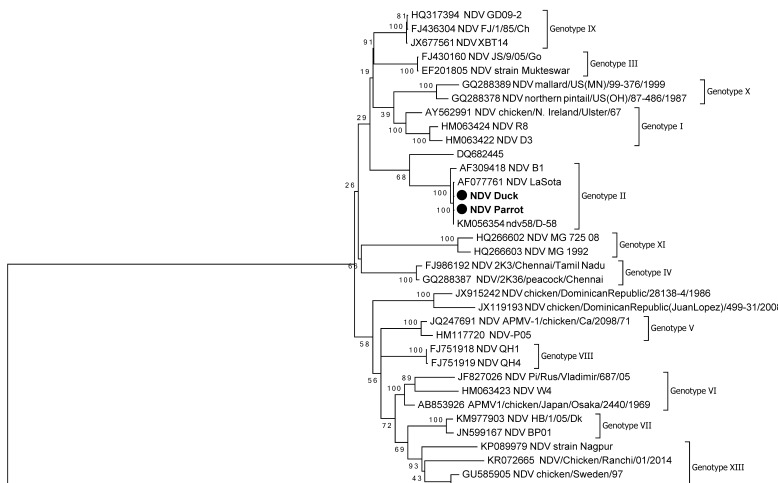
Phylogenetic tree constructed based on full-length *F* gene sequence of the NDV isolates. The tree was constructed using the neighbor-joining algorithm of MEGA X (1000 bootstrap repetition). The NDV duck (As/Km/19/44, GenBank acc. MZ318446) and parrot (As/WB/19/91, GenBank acc. MZ318447) designated (●) were compared to commercial vaccine strains LaSota (GenBank acc. AF077761) and B1 (GenBank acc. AF309418) obtained from the GenBank.

The phylogenetic tree was constructed based on the full-length *F* gene sequences of the NDV isolates from duck and parrot (Figure 4). The nucleotide sequence of our isolate showed the highest identification, 99.88 to 99.94%, with a recently reported genotype II ndv58/D-58 strain of NDV isolated from chicken, India (GenBank acc. KM056354). Phylogenetic analysis with reference vaccine strains LaSota (GenBank acc. AF077761) and B1 (GenBank acc. AF309418) revealed that both the NDV isolates from duck and parrot have nucleotide similarity of upto 99.81 to 99.87% and 98.89 to 98.98%, respectively. The isolates in this study also had similarities with SD/5/04/Go strain of NDV isolated from goose, China (GenBank acc. DQ682445) of 91.66 to 91.78%. The GenBank acc. numbers of isolates in this study are MZ318446 (As/Km/19/44) and MZ318447 (As/WB/19/91).

### 3.2. Adaptation of As/Km/19/44 and As/WB/19/91 in Chicken

The NDV isolates from duck (AS/Km/19/44) and parrot (As/WB/19/91) were serially passaged in SPF chicks in order to facilitate adaptation in chicken. After the six passages, chicken-adapted NDV from duck (PSD44C) and parrot (PSP91C) were recovered from the inoculated chicks and propagated in ECE for further use.

### 3.3. Biological Pathogenicity Assessment of Chicken-Adapted PSD44C and PSP91C

The MDT of PSD44C and PSP91C was 116.50 ± 4.646 and 95. 25 ± 2.868 and its ICPI was 0.28 ± 0.039 and 0.453 ± 0.028, respectively. Both the isolates were confirmed as lentogenic strains, according to their MDT and ICPI values (Table 1).

### 3.4. Thermostability Profile of Chicken-Adapted PSD44C and PSP91C at 40 °C and 56 °C

**Figure 5:**
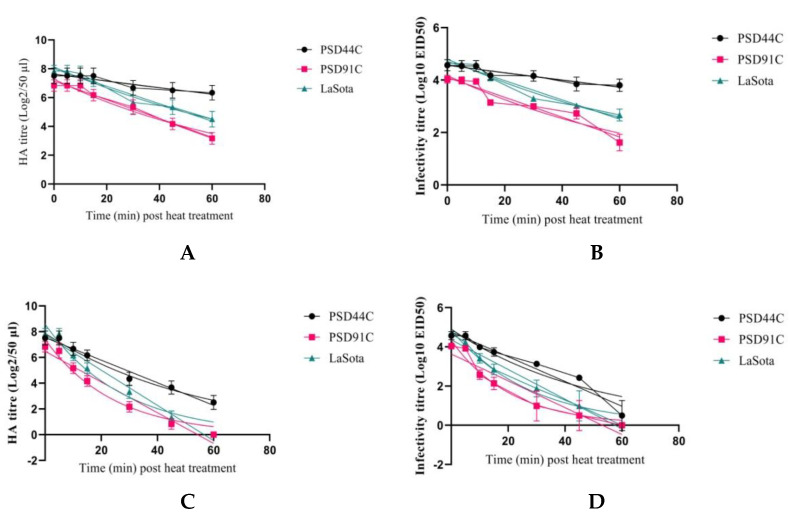
Hemagglutinin (HA) stability (Log_2_HA) and infectivity titer (Log_10_EID_50_) of chicken-adapted PSD44C, PSP91C and LaSota strain at different temperature-time intervals ((**A**): HA activity at 40 °C, (**B**): Infectivity at 40 °C, (**C**): HA activity at 56 °C, (**D**): Infectivity at 56 °C).

The results of thermostability testing and half life values of PSD44C, PSP91C and LaSota at 40 °C and 56 °C are tabulated in Table 2 and the degradation curve for each of the NDV isolates is depicted in Figure 5. The results showed that biological decay of virus titer is variable for PSD44C, PSP91C and LaSota over different temperatures.

#### 3.4.1. Thermostability Testing at 40 °C

The initial HA titer (Log2/50 µL) and infectivity (Log_10_EID_50_) of PSD44C, PSP91C and LaSota were (7.636, 4.572), (7.274, 4.175) and (8.125, 4.801), respectively. PSD44C retained its HA activity and infectivity for an extended period of time and resulted in a half life of 206.50 and 205.00 min, respectively, as compared to PSP91C (56.91, 55.51 min) and LaSota (70.42, 67.016 min).

#### 3.4.2. Thermostability Testing at 56 °C

The initial HA titer (Log2/50 µL) and infectivity (Log_10_EID_50_) of PSD44C, PSP91C and LaSotawere (7.869, 4.902), (7.402, 4.334) and (8.582, 4.860), respectively. PSD44C retained its HA activity and infectivity for an extended period of time and resulted in a half life of 38.39 and 34.36 min, respectively, as compared to PSP91C (16.79 and 14.59 min) and LaSota (19.20 and 19.19 min).

Based on these results, the degradation curves of PSP91C exhibited more rapid decay of HA activity and infectivity at each point of time followed by LaSotaat 40 °C and 56 °C.

### 3.5. Immunogenicity of the Chicken-Adapted PSD44C and PSP91C

Upon inoculation with ten times the single dose of both the chicken-adapted PSD44C and PSP91C intraocularly, the chicks did not show any local and/or systemic adverse reactions or death throughout the observation period of 21 days, revealing the isolates to be safe for immunization studies. Before immunization, chicks of all study groups were sero-negative. Post immunization, the chicks of groups T_1_ and T_3_ immunized with PSD44C and LaSota vaccine strains, respectively, showed significantly higher HI antibody titer (*p* < 0.0001) as compared to group T_2_ immunized with PSDP91C at different dpi (Table 3 and Table S1). At 7 dpi, there was a rise in the HI (log_2_) antibody levels of all the chicks immunized with PSD44C, T_1_ (3.20 ± 0.14), PSP91C, T_2_ (2.30 ± 0.11) and LaSota vaccine strain, T_3_ (3.30 ± 0.11). In all the three immunized groups, the HI (log_2_) antibody titer values increased steadily with each day and reached the titer peak at 21 dpi (T_1_: 7.10 ± 0.12; T_2_: 4.10 ± 0.0.16; T3: 7.25 ± 0.14) which were maintained up to the end of the study period of 28 days (T_1_: 6.90 ± 0.12; T_2_: 4.000 ± 0.18; T3: 7.000 ± 0.15). In the present study, there were no postvaccinal reactions in the immunized groups. Taken together, our results reflected that PSD44C is a better immunogenic than PSP91C and displayed a similar antibody profile as the commercial LaSota vaccine strain in chickens.

## 4. Discussion

The lentogenic NDV strains from class I and genotypes I and II of class II are commonly isolated from apparently healthy domestic poultry and wild birds [20,21,22,23,24,25]. There are also reports on the lentogenic NDV strains isolated from parrots [26,27,28]. In this study, we characterized NDV isolates from cloacal swab and intestinal content of duck and parrot. In this study, molecular characterization of NDV isolates from duck and parrot revealed class II genotype II NDV. Based on the analysis of the F protein cleavage site, both the isolates shared the cleavage site motif ^112^GRQGRL^117^ with an amino acid residue leucine (L) at position 117 which is a typical feature of lentogenic strain [13]. The F cleavage site has been shown to be the most important determinant of NDV virulence [29]. The sequences recovered in this study were compared with class II genotype II NDV sequences previously deposited in GenBank and reference vaccine strains LaSota and B1. The genome sequences of both duck (99.88 to 99.94%) and parrot (91.66 to 91.78%) showed similarity to NDV strains isolated from chicken, India (GenBank acc. KM056354) and goose, China (GenBank acc. DQ682445), respectively. Similar findings of the presence of class II NDV in ducks in South Korea and China were reported [30,31]. Analysis of identity showed that isolates recovered in this study weremost closely related to a recently reported genotype II ndv58/D-58 strain of NDV isolated from chicken, India (GenBank acc. KM056354).

The European Union stated in their Commission Decision 93/152/EEC that for routine ND vaccination programs the viruses used as live NDV vaccines were to be tested under specific conditions and have an ICPI of less than 0.4 or 0.5, depending on the dose of vaccine given. Similarly, the OIE Biological Standards Commission recommended in 2000 that principal vaccines should have an ICPI <0.7. OIE proclaimed that the first principle to consider when selecting a strain for a live NDV vaccine is to determine its pathogenicity. In the present study, the biological pathogenicity assessment of the origin of chicken-adapted NDV isolates of duck (PSD44C) and parrot (PSP91C) showed that both the isolates were lentogenic with MDT more than 90 h and ICPI ranging from 0.2 to 0.4. Earlier, pathogenicity assay-like MDT in eggs and the intravenous pathogenicity test (IVPI) have been used [32], but the OIE defines the assessment of virus virulence based on the ICPI. Further, our study indicates that the chicken-adapted virus did not acquire virulence after serial back passages.

Vaccination is one of the effective measures to curb the economic menace that is ND. Despite immunization regimens against ND using live vaccines or both live and inactivated vaccines [33], NDV continues to impact the poultry industry. Almost all available commercial vaccines like B1 and LaSota are thermolabile, and subsequently require a cold chain to retain their potency [28,34]. Thermostable vaccines help to ensure vaccine potency with minimum dependence on cold chain. Several thermostable strains such as V4, HR-V4 and I2 have been isolated and characterized [35,36,37]. Since then, several techniques have been employed to select thermostable strains from a heterogenous population of viruses [28,38,39]. This study profiled the thermostability of the lentogenic chicken-adapted NDV isolates from duck (PSD44C) and parrot (PSP91C). The results showed that the temperature-dependent biological decay of the virus titer was variable for both the isolates. PSD44C retained its HA activity and infectivity for an extended period of time and resulted in a half life of 206.50 and 205.00 min, respectively, at 40 °C as compared to PSP91C (56.91 and 55.51 min) and LaSota (70.42 and 67.016 min). The chicken-adapted PSD44C isolate could withstand the thermal exposure of 56 °C for a half life of 38.39 and 34.36 minfor the HA and infectivity titers, respectively, whereas PSP91C (16.79 and 14.59 min) and LaSota (19.20 and 19.19 min) had shorter half lives. Literature in this aspect is scant. However, Li et al. [40] reported thermostable avirulent NDV strain D4 from duck in China. In contrast, thermostability testing of 100 lentogenic field isolates from Germany revealed thermolabile hemagglutinins [41]. The degradation curves of PSP91C exhibited more rapid linear decay of HA activity and infectivity at each point of time when exposed to 40 °C and 56 °C, followed by LaSota which might be due to the destruction of hemagglutinin spikes of the virus on exposure to higher temperatures, leading to the inability of the virus to agglutinate chicken red blood cells as well as loss of infectivity [6]. Therefore, it is evident from our results that the chicken-adapted PSD44C is comparatively more thermostable than PSP91C and LaSota.Previous studies on the evaluation of thermostability profile of commercially available vaccine strains B1 and LaSota reported them to be thermosensitive [28,34]. This is in agreement with our results. A study reported that the lentogenic Dongola strain of NDV maintained the stability of hemagglutinins and infectivity up to 15 min after exposure at 56 °C [42]. Avirulent NDV strains with a thermostable hemagglutinin were found in isolates from migrating geese [43]. From our study, it can be concluded that field isolates displayed a source of avirulent NDV with varied thermostability profile. Further, with a degree of improvement concerning thermostability the chicken-adapted NDV isolate from duck could provide a promising line of research in the development of thermostable vaccine. Thermostability studies suggest that heat-stable hemagglutinins are derivable through a series of selection procedures of relatively thermostable progeny virus, rather than the parent virus [34,44]. In the present study, both the chicken-adapted PSD44C (Log_2_5.73 ± 0.18) and commercial LaSota vaccine strain (Log_2_5.86 ± 0.19) elicited a significantly (*p* < 0.0001) higher antibody response than the chicken-adapted PSP91C (Log_2_3.45 ± 0.11) in chicken. The OIE defines a “protective” antibody titer as Log_2_ 4 or more against 4HAU of antigen. Thus, PSD44C might be a better thermostable immunogen in comparison to PSP91C and LaSota vaccine strain.

## 5. Conclusions

In the present study, the thermostability of the chicken-adapted NDV isolates from duck (PSD44C) and parrot (PSP91C) was evaluated at 40 °C and 56 °C, and compared with commercially available LaSota vaccine strain on the basis of cumulative infectious titer drop and HA activity. Our results showed that PSD44C was the most thermostable as compared to PSP91C and LaSota vaccine strain. Further, the immunogenicity of PSD44C and LaSota was significantly higher than PSP91C. Therefore, it is concluded that NDV isolate PSD44C is more thermostable and immunogenic when administered intraocularly without any adverse effects and is suitable for further research and vaccine development.

## Figures and Tables

**Table 1:** Pathogenicity assessment of chicken-adapted Newcastle disease virus (NDV) isolates PSD44C and PSP91C.

NDV Isolates	MDT ^a^ in Hours (n = 4) (Mean ± SE)	ICPI ^b^ (*n* = 4)	Pathotype
PSD44C	116.50 ± 4.646	0.28 ± 0.039	Lentogenic
PSP91C	95. 25± 2.868	0.453 ± 0.028	Lentogenic

^a^ Mean Death Time, ^b^ Intra Cerebral Pathogenicity Indices.

**Table 2:** Comparison of degradation values of PSD44C, PSP91C and LaSota exposed at 40 °C and 56 °C for different time points.

Temperature (°C)	NDV Strain	Initial Titer		Regression Analysis				Half Life (Minutes)	
		HA Titer (Log_2_/50 µL)	Infectivity Log_10_EID_50_	HA Activity	Rate Constant (K)	Infectivity	Rate Constant (K)	HA Activity	Infectivity
40 °C	PSD44C	7.636	4.572	Y= −0.02321X + 7.618	0.003357	Y =−0.01392X + 4.561	0.003381	206.50	205.00
	PSP91C	7.274	4.175	Y= −0.06519X + 7.156	0.01218	Y = −0.03785X + 4.099	0.01249	56.91	55.51
	LaSota	8.125	4.801	Y= −0.06061X + 8.000	0.009842	Y = −0.03675X + 4.714	0.01032	70.42	67.016
56 °C	PSD44C	7.869	4.902	Y= −0.08772X + 7.544	0.01806	Y = −0.06316X + 4.761	0.02017	38.39	34.36
	PSP91C	7.402	4.334	Y= −0.1192X + 6.476	0.04129	Y = −0.06840X + 3.634	0.04750	16.79	14.59
	LaSota	8.582	4.860	Y= −0.1367X + 7.746	0.03610	Y = −0.07565X + 4.358	0.03612	19.20	19.19

**Table 3:** Serum antibody titers (Mean ± SE) in chicks against the NDV isolates used for immunization: (T_1_) chicken-adapted NDV of duck origin (PSD44C), (T_2_) chicken-adapted NDV of parrot origin (PSP91C) and (T_3_) Control (LaSota vaccine strain).

Treatment Groups *	Days Post Immunization (DPI) **					Overall
	0	7	14	21	28	
T_1_	0.00 a^A^	3.20 ± 0.14 _b_^B^	5.70 ± 0.11 _c_^B^	7.10 ± 0.12 _d_^B^	6.90 ± 0.12 _d_^B^	5.73 ± 0.18 ^A^
T_2_	0.00 a^A^	2.30 ± 0.11 _b_^C^	3.40 ± 0.11 _c_^C^	4.10 ± 0.16 _d_^C^	4.00 ± 0.18 _d_^C^	3.45 ± 0.11 ^B^
T_3_	0.00 a^A^	3.30 ± 0.11 _b_^B^	5.90 ± 0.07 _c_^B^	7.25 ± 0.14 _d_^B^	7.00 ± 0.15 _d_^B^	5.86 ± 0.19 ^A^

* Means with different superscripts within a column and subscripts within a row differ significantly. ** HI (log_2_) antibody titer in sera of immunized chicks at different days post immunization (dpi).

## Data Availability

Not applicable.

## Supplementary Materials

The following supporting information can be downloaded at: https://www.mdpi.com/article/10.3390/v14112528/s1, Table S1: ANOVA of serum antibody titers in chicks against NDV isolates used for immunization showing the effects of treatment and period.
