# Supplementary material for: Thermostability and Immunogenicity of Genotype II Avian Orthoavulavirus (AOaV-1) Isolates from Duck (Anas platyrhynchos) and Parrot (Eclectusroratus)
Source: Viruses. 2022 Nov 15;14(11):2528. doi: 10.3390/v14112528 (PMC9697861; doi:10.3390/v14112528)
Supplement: Supplementary file 1 [file viruses-14-02528-s001.zip › viruses-1937375-supplementary.pdf]

Supplementary Materials

Table S1: ANOVA of serum antibody titers in chicks against NDV isolates used for immunization showing the effects of treatment and period

Source of variation	DF	SS	MS	F	*p* value
Interaction	8	48.39	6.049	449.6****	$p < 0.0001$
Time	4	798.8	199.7	14844****	$p < 0.0001$
Treatment	2	117.5	58.75	4367****	$p < 0.0001$
Residual	135	1.816	0.01345		

**** Highly significant ($p < 0.0001$)
